# Millimeter-Wave Imaging System Based on Direct-Conversion Focal-Plane Array Receiver

**DOI:** 10.3390/s22197132

**Published:** 2022-09-20

**Authors:** Sergey Korolyov, Aleksandr Goryunov, Ivan Illarionov, Vladimir Parshin, Petr Zemlyanukha

**Affiliations:** 1 Institute for Physics of Microstructures of the Russian Academy of Sciences, 603950 Nizhny Novgorod, Russia; 2 Institute of Radio Electronics and Information Technologies, Nizhny Novgorod State Technical University, 603950 Nizhny Novgorod, Russia; 3 Institute of Applied Physics of the Russian Academy of Sciences, 603950 Nizhny Novgorod, Russia

**Keywords:** millimeter-wave imaging, frequency-modulated continuous-wave (FMCW) radar, focal-plane array (FPA), direct-conversion receiver, low-barrier diode

## Abstract

A new approach to millimeter-wave imaging was suggested and experimentally studied. This approach can be considered as the evolution of the well-established focal-plane array (FPA) millimeter-wave imaging. The significant difference is the use of a direct-conversion array receiver, instead of the direct-detection array receiver, along with the frequency-modulated continuous-wave (FMCW) radar technique. The sensitivity of the direct-conversion receiver is several orders higher than the sensitivity of the direct-detection one, which allows us to increase the maximum imaging range by more than one order of magnitude. The additional advantage of the direct-conversion technique is the opportunity to obtain information about the range to an object. The realization of the direct-conversion FPA imaging system was made possible due to original sensitive simple-designed receiving elements based on low-barrier Mott diodes. The suggested imaging method’s main characteristics, which include the achievable angular and range resolution and the achievable maximum imaging range, were studied. A maximum range of up to 100 m was experimentally determined. A 94 GHz 8 × 8 imaging system was developed for demonstration purposes and studied in detail. The suggested technique is assumed to be useful for creating a long-range millimeter-wave camera, in particular, for robotic systems that operate in poor environmental conditions.

## 1. Introduction

Millimeter-wave (MMW) range (30–300 GHz) is currently considered to be an optimal radio frequency (RF) sub-band for imaging [1,2,3,4]. MMW imaging systems combine high resolution with low absorption in air and many other mediums and materials. The ability of MMW radars to enable the visibility of objects in fire environments was proven [5]. The MMW band is demonstrated to be an ideal part of the electromagnetic radiation to diagnose human skin conditions [6]. Terahertz (0.3–3 THz) imaging is being actively developed for short-range applications [7].

A lot of methods of MMW imaging have been proposed to date. A mechanical scanning is conceptually the simplest one. In this method, an image can be built up by scanning a single pixel receiver, either by moving a receiver in the focal plane of the antenna or mechanically scanning the beam direction of the system [1]. A shortcoming of the single-element mechanical scanning is a low frame rate. For increasing the speed, a receiver of the mechanically scanning systems can consist of several sensing elements (usually, <~10) [8,9,10,11]. A real-time passive imaging is achieved in these systems [8]. A further increase of a number of the sensing elements in the receiving array leads to emerging qualitatively new types of imaging methods that cannot include the mechanical movements at all. These methods make it possible to create imaging systems with higher sensitivity and portability.

The interferometric array, the phased array, and the focal-plane array (FPA) are the main types of the multi-element imaging systems [1]. The interferometric [12] and phased array [13] systems are based on measuring or manipulating the phase of the signal, which requires the creation of the complicated array RF circuits. The multiple-input/multiple-output (MIMO) radar technique [14] allows us to reduce a total number of the interferometric array elements, and this is achieved by using a number of transmitting elements that radiate orthogonal waveforms. This makes the interferometric systems more applicable. A number of the MIMO radar systems have been presented to date [15,16]. In Reference [17], a high-resolution MMW imaging system combining 2D MIMO arrays with synthetic aperture radar (SAR) technique, along with a novel Fourier-based image reconstruction algorithm using sparsely sampled aperture data, was proposed.

Special attention is paid to the development of optimal signal-processing methods and algorithms for MMW imaging. Recently, algorithms for fast fully focused imaging, which includes the case of location-constrained MIMO arrays or partially damaged transceivers, were proposed [18,19]. Methods for improving the resolution of the passive MMW images and making it more preferable for object recognition were suggested and studied [20,21]. A method that allows detecting > 15 distinct skeletal joints by using MMW radar reflection signals was developed [22].

In FPA imaging systems, an image is built up with a quasi-optical objective, and direct-detection receiving elements can be used for measuring RF power distribution at the focal plane [1,2,23]. The FPA approach can be applied to both passive [2] and active [23] imaging. In passive mode, a role of the radiation sources plays the imaged objects themselves; thus, the received power is proportional to 1/*r*^2^, where *r* is the range to an object. In the active mode, the received power is proportional to 1/*r*^4^. At a typical power level of the MMW sources (~1–100 mW), the maximum range of the active imaging systems is usually not more than several meters.

According to the classic radar equation [13], the maximum range of the active FPA system can be increased in several ways. The first way is an increase in the gain of the transmitting antenna. This approach, however, leads to a reduction in the beamwidth of the transmitting power and, as a consequence, to a reduction in the field of view of the imaging system. The maximum range can be enhanced by increasing the quasi-optical objective aperture. The undesirable result of this approach is an increase in the system’s dimensions. The third way is to enhance power of the RF source. This way, however, does not look attractive: the power should be increased ten thousand times for a tenfold increase in the maximum range. The fourth way is to enhance the sensitivity of the receiver. A more sensitive FPA receiver can be built by using the heterodyne-detection principle that is based on mixing the received signal with the reference one. The magnitude of the output signal in this case is proportional not to the RF power but to the amplitude of the RF field, which depends on the range to a target as 1/*r*^2^. The heterodyne-type receiving allows not only for a significant increase in sensitivity of the imaging systems, but also provides the ability to employ radar methods. It becomes possible to determine the range to a target and its radial velocity in addition to a 2D picture of the scene.

The superheterodyne-type FPA imaging systems were studied [1,24,25], but they have not been widely used. The main reason for this is difficulties in creating an array with a high number (>~100) of the receiving elements. Feed of local oscillator (LO) power to the array elements, reduction in the power consumption, and decrease in the cost are some of the difficulties to be overcome.

In this work, we suggest a new approach to creating FPA imaging system that we studied. This approach is based on employing a direct-conversion array receiver, which has a simpler design in comparison to the superheterodyne receiver and, at the same time, can achieve higher sensitivity in comparison to the direct-detection receiver. Realization of the suggested approach became possible due to developed sensitive receiving elements that could be relatively easily integrated into the multi-element array [26].

## 2. Imaging Principle

**Figure 1:**
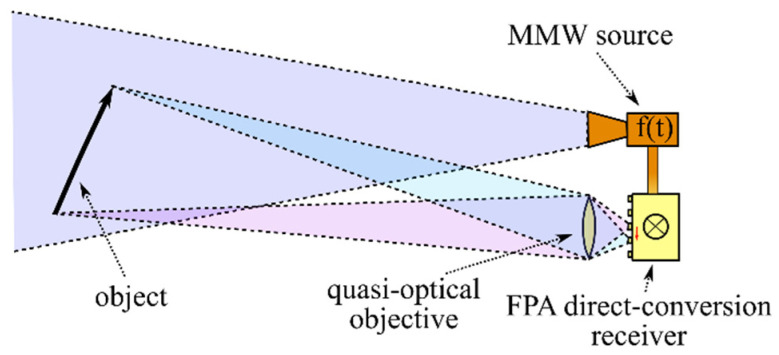
A general representation of the imaging principle. A 2D image is formed by a quasi-optical objective, and the radial coordinate is determined by analyzing the phase of the beat frequency (BF) signal.

An operation principle of the suggested imaging system is generally depicted in Figure 1. An MMW source provides a frequency-modulated (FM) continuous wave, the power of which is divided into two parts. The first part goes to illuminating the scene, and the second part goes to the receiver as the LO power. An object (or objects) at the scene scatters the incident power in different directions. A part of the power reflects toward a quasi-optical objective that focuses it at the focal plane. Each point of the focal plane corresponds to the direction that the signal comes from. The focused radiation is captured by antennas of the array, and, at each pixel, the received RF signal is mixed with the LO signal. The frequency modulation of the MMW signal leads to that the frequency of the echo signal differs from the frequency of the LO signal, which results in appearance of a beat frequency (BF) signal at the output of the mixing element. Distribution of the BF signal amplitude among the array elements corresponds to the distribution of reflectors over the scene. Information about the radial coordinate and velocity can be evaluated from the phase of the BF signal in accordance with the FM continuous-wave (FMCW) radar technique [27,28,29].

As described above, a 2D image and the radial coordinates of the objects are obtained in different ways. The 2D image is formed by the quasi-optical objective. In accordance with the laws of geometrical optics [30], the surface RF power density (*ρ_RF_*) at a point of the focal plane *P* with coordinates (*ξ*, *η*) is related with the radiant intensity (*I_Ω,RF_*) incident onto the objective from the direction $\vec{n}$ with coordinates (*α*, *β*) as follows:

(1) $$\rho_{RF}\left(\xi \left(\alpha ,\beta \right),\eta \left(\alpha ,\beta \right)\right)\;=\;\frac{I_{\Omega ,RF}\left(\alpha ,\beta \right)}{\partial \left(\xi ,\eta \right)/\partial \left(\alpha ,\beta \right)},$$

where ∂(*ξ*,*η*)/∂(*α*,*β*) is the Jacobian determinant.

The radial coordinate (*r*) of the object is related to the phase of the received RF signal [27]:

(2) $$\varphi_{RF}\left(\xi \left(\alpha ,\beta \right),\eta \left(\alpha ,\beta \right),r\right)\;=\;2\pi \int_{0}^{t-2r/c}fdt,$$

where *f* is the frequency of the MMW signal, *c* is the velocity of propagation, and *t* is the time. As can be seen from (2), measurement of the phase may allow determining not only the range to an object but the 3D image entirely.

The received RF signal at the focal plane has the surface power density given by (1) and the phase given by (2). Let us denote *P_RF_*($\Delta {\vec{n}}_{i}$) as the power that falls onto the area Δ*P_i_* of the array element number *i*, where $\Delta {\vec{n}}_{i}$ is a solid angle of the object space that corresponds to Δ*P_i_*. The phase of the incident signal is denoted by *Φ_RF_*($\Delta {\vec{n}}_{i}$, *r*). The integrated quantities {*P_RF_*, *Φ_RF_*} are determined by the differential quantities {*ρ_RF_*, *φ_RF_*} and parameters of the antenna array.

The RF signal is mixed with the LO signal that has the power per element, *P_LO_*, and the phase, *Φ_LO_*. The resulting BF current is as follows [26,27]:

(3) $$\;i_{BF}\left(\Delta {\vec{n}}_{i},r\right)\;=\;\Sigma \left(P_{LO}\right)\sqrt{P_{RF}\left(\Delta {\vec{n}}_{i}\right)}cos\left(\Phi_{RF}\left(\Delta {\vec{n}}_{i},r\right)-\Phi_{LO}\right),$$

where *Σ*, which is dependent on *P_LO_*, characterizes the sensitivity of the mixing element.

As can be seen from (3), the amplitude of the BF current is *I_BF_*~*P_RF_*^1/2^; the BF is *f_b_*~|*f*(*t* − 2*r*/*c*) − *f*(*t*)|, which can be obtained by taking into account (2). At a linear dependence of the MMW signal frequency on time (*f*~*t*), the BF is *f_b_*~*r*.

## 3. 1 × 3 Array Imaging System

**Figure 2:**
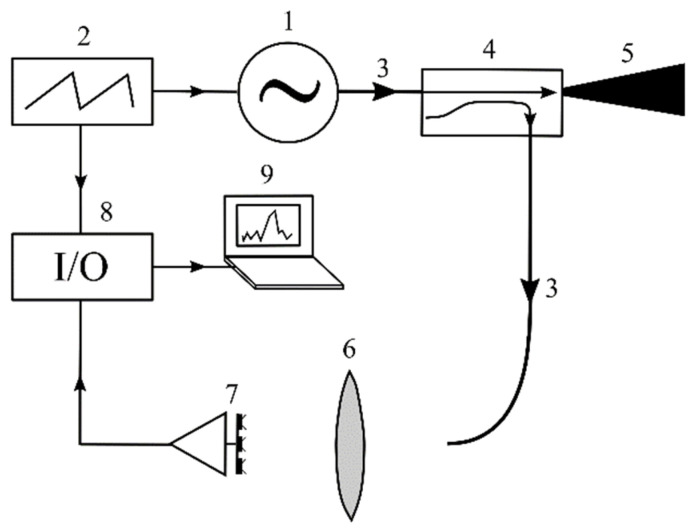
A schematic representation of the 94 GHz imaging system with a direct-conversion 1 × 3 FPA receiver. (1) W-band BWO, (2) function generator, (3) rectangular waveguides, (4) directional coupler, (5) horn antenna, (6) dielectric lens, (7) array receiver, (8) I/O device, and (9) laptop.

A 94 GHz imaging system with a direct-conversion 1 × 3 FPA receiver was developed for studying the basic characteristics of the suggested imaging method. A schematic representation of the imaging system is shown in Figure 2. An RPC “Istok” OV-71 backward-wave oscillator (BWO) is used as the W-band MMW source. The BWO output power weakly depends on the frequency in the range from 82 to 110 GHz. A value of BWO power near 94 GHz is about 10 mW. The frequency modulation of the oscillator is performed with a LG FG-8002 function generator by changing the BWO anode voltage near the operating point. A dependence of the frequency from the anode voltage has a quasilinear form in the operating range with a slope of 48 MHz/V. The chirp modulation waveform is used with a period of 4.5 ms and peak-to-peak amplitude of 12 V, which corresponds to a bandwidth of *B* = 576 MHz.

The radiation of the source passes through a metal waveguide to a Quinstar QJG-E03300 directional coupler, where the power is almost equally divided into two parts. One of these parts is used to illuminate the scene with a conical horn antenna, which has a directivity of about 26 dB. The other part of the power passes to the receiver as LO power.

An air path is used to provide LO power to the receiver. The LO power is radiated from the open end of the waveguide that is located at the focus of a dielectric lens. The radiation of a point source is collimated with the lens to a parallel beam, which falls on the array receiver.

A high-density polyethylene (HDPE) aspherical lens with a diameter of 10 cm is used as a quasi-optical objective for focusing received RF power and collimating the LO power. The focal length of the lens is 10 cm. The lens is not symmetrical relatively to its plane; a side facing the scene is more convex. The asymmetrical aspherical shape provides low aberration, hence the small size of the focal spot. A spot size of about 4 mm was experimentally evaluated [31], which makes it possible to achieve high resolution of imaging.

**Figure 3:**
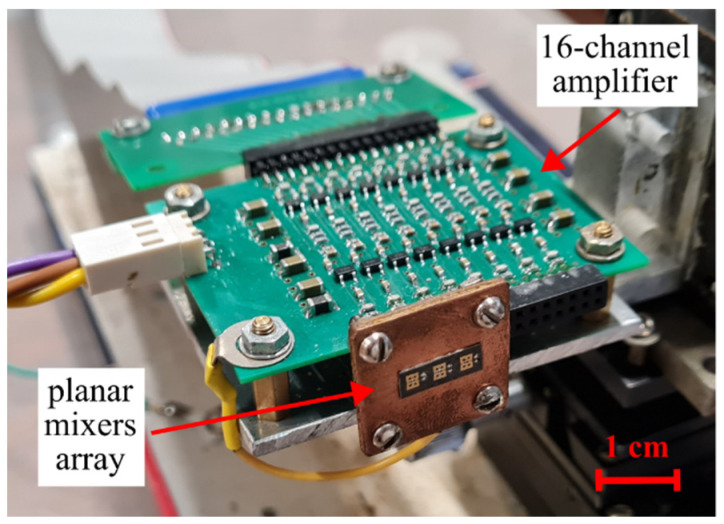
A photograph of the 1 × 3 array receiver. The receiver consists of a planar mixers array and a multi-channel transimpedance amplifier.

A three-element array receiver is located at the focal plane of the lens. A photograph of the receiver is shown in Figure 3. Three modified slot antennas are formed on a Rogers 5880 dielectric plate by photolithography methods. A low-barrier Mott diode [32] that serves as a mixing element is mounted on each antenna with conductive glue. The diodes operate at zero bias, and therefore the RF front-end of the receiver does not require any power supply. Low-barrier Mott diodes with a differential resistance (*R_d_*) of about 3 kΩ were chosen, which corresponds to the minimum value of the effective barrier height (~0.2 eV) that can be controllably achieved within the developed technology. The curvature coefficient (*α* = *R_d_*·d^2^*i*/d*u*^2^), which is determined from the dc current–voltage characteristic *i*(*u*) of the diodes, is about four times less than for an ideal Mott diode (*α_ideal_* = 40 A/W), which is related with effects of lowering the barrier height. The capacitance of the diodes is about 15 fF, and the series resistance is 15–40 Ω [33].

The dielectric plate with receiving elements is attached to a copper-coated substrate that provides rigidity of the module. The BF signal is output from the diodes with thin wires that are routed from the antennas and leaves through the dielectric plate to the rear side of the substrate, where connectors for the BF amplifier are mounted.

The BF current of the diode is converted to voltage via a transimpedance amplifier that has a gain of 110 kΩ and a frequency band from 100 Hz to 100 kHz. A compact 16-channel transimpedance amplifier was developed for creating an 8 × 8 array receiver (see Section 5). For the 1 × 3 array, 3 of 16 channels were used.

The sensitivity of the developed 3-element array receiver was studied in [34]. It was found that the noise equivalent power (NEP) of the direct-conversion receiving elements at an accessible LO power of 10 μW per element is about 10^−17^ W/Hz. The estimations show that the found sensitivity value corresponds to increasing the maximum range of the FPA imaging system by 30 times, if comparing with the direct-detection receivers. In this work, the estimations of [34] were confirmed experimentally.

The BF signals of the receiving elements pass to an NI USB-6225 multifunction I/O device, which is synchronized with the function generator. The signals are sequentially digitized and written to the buffer during 10 periods of modulation for each channel. The signal processing is performed on a laptop that obtains the data from the I/O device buffer with a USB port. The data of each channel are averaged over the 10 periods of modulation, which were written in one cycle. The averaged signals undergo a fast Fourier transform (FFT) procedure, resulting in the amplitude spectrums of the BF signals of all receiving elements.

## 4. Basic Characteristics of the Imaging Method

The 1 × 3 array imaging system was developed for studying the main characteristics of the suggested imaging method. The resolution and the maximum imaging range were part of the focus of our exploration.

### 4.1. Resolution

The suggested method has a possibility of acquiring a 3D image of the scene. As it is described in Section 2, a 2D image is formed via the quasi-optical objective, whereas distance information is obtained by using the FMCW radar technique. Thus, two parameters that characterize the resolution of the system should be considered: the angular and range resolutions, respectively.

**Figure 4:**
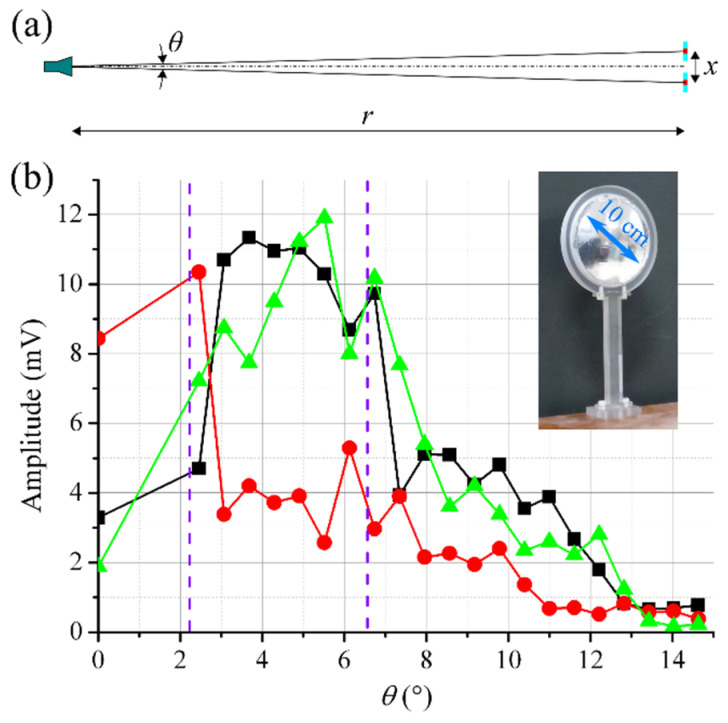
(**a**) A disposition scheme of the imaging system and the reflectors in an experiment of angular resolution study. A photo of the reflector is in the inset. (**b**) The amplitude of the BF signals of the left (black), the central (red), and the right (green) receiving elements in dependence on the angle between the reflectors (*θ*). The order of the receiving elements corresponds to Figure 3. The vertical violet lines in the graph are placed at the angles at which the reflected RF power falls onto boundaries of the receiving elements.

The main geometric parameters of the experiment on measuring the angular resolution of the imaging system are shown in Figure 4a. Two identical reflectors are placed at the distance *r* = 374 cm from an objective of the imaging system. A photograph of the reflector is shown in the inset of Figure 4. The convex metalized surface of the reflector has a size of about 10 cm. The RF power is reflected to the objective from a small central area (~1–2 cm) of the metalized surface that is schematically marked in red in Figure 4a. Thus, a size of the reflecting area is much less than a size of a space region corresponding to a field of view of one array element (~15 cm at *r* = 374 cm).

The amplitude of the BF signals of the receiving elements is measured in dependence on the spacing between the reflectors (*x*), which can be easily recalculated to an angle between directions from the objective to the reflectors (*θ*). The measurement data are presented in Figure 4b. The value *x* = 0 (*θ* = 0) corresponds to the maximum amplitude of the signal of the central receiving element. One reflector was used for obtaining measurement data at this point. The next value of *x* (*θ*), at which the measurement was performed, corresponds to the minimal possible spacing between two reflectors (*x* = 16 cm, and *θ* = 2.5°). Then the measurements were carried out with a step of 4 cm.

At *θ* ≤ 2.5°, the RF power reflected from the metalized surfaces of the reflectors is focused only on the central receiving element. The signals of the side elements are caused by clutter reflections from the frames of the reflectors. Then, at 2.5° < *θ* < 3.1°, the amplitude of the BF signals of the side receiving elements increases, while the amplitude of the central receiving element decreases. This is caused by RF power focused with the lens crossing the boundaries between the central and side receiving elements. A value of *θ* that corresponds to the positions of these boundaries is shown by a vertical dashed line in Figure 4b (the left line). At *θ* = 3.1°, the amplitude of the side receiving elements is about two or three times greater than the amplitude of the central receiving element. Thus, the angular resolution of not worse than 3° can be established. At 6.7° < *θ* < 8.0°, spots of the focused RF power leave the array region, the boundaries of which correspond to the right vertical dashed line in Figure 4b.

The range resolution was studied by using the similar disposition of the imaging system and the reflectors, as in Figure 4a. A difference was that the reflectors were placed at a constant (minimum possible) angular spacing, and the radial distance (*z*) between them was variable (one of the reflectors was moved toward the imaging system). The central receiving element was used in this experiment. A value of *z* = 30 cm, at which the reflectors began to become distinguishable, was obtained. This value is in good agreement with a value of *δr* = 26 cm that is obtained from a well-known expression for the range resolution of an FMCW radar system *δr* = *c*/2*B*.

### 4.2. Maximum Range

**Figure 5:**
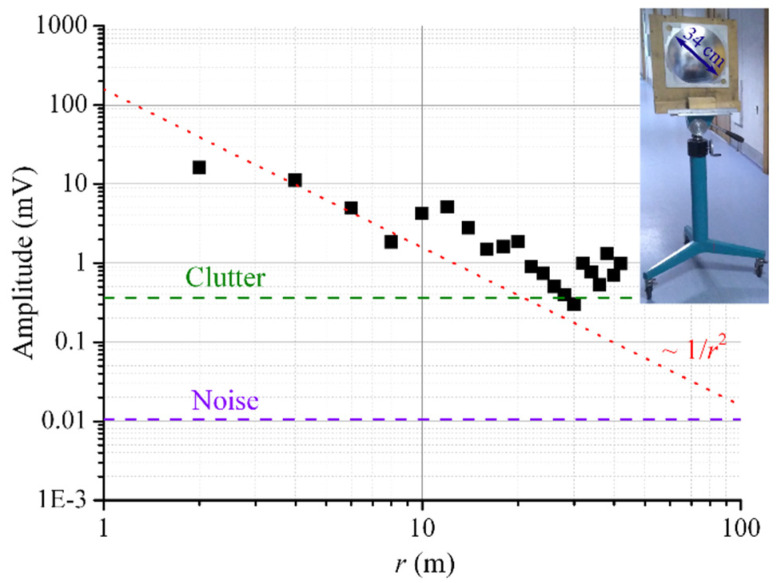
The amplitude of the BF signal in dependence on the range to a reflector (*r*). The black points are the experimental data; the red dotted line is an approximation by the function *C*/*r*^2^, where *C* is a fitting parameter. The green dashed line is the level of the clutter; the violet dashed line is the level of the receiver noise. Inset: a photograph of the reflector.

The maximum range of the imaging system was studied in a long hall. The imaging system was placed at one end of the hall, and a reflector was moved from the imaging system to the other end of the hall with a movable tripod. A photograph of the reflector mounted on the tripod is shown in the inset of Figure 5. The diameter of the metalized diffuse surface of the reflector is 34 cm. The amplitude spectra of the BF signal of the central receiving element were recorded at each step of the movement.

Values of the BF signal amplitude obtained at different reflector ranges are represented by black dots in Figure 5. The experimental data are in a good agreement with the theoretically predicted dependence of the amplitude on the range *C*/*r*^2^, where *C* is a fitting parameter. An approximation by this function is shown by a red dotted line in Figure 5. The experimental data were obtained up to a range of about 40 m, which was restricted by a clutter signal (a green dashed line in Figure 5) caused by reflections from the walls, floor, and ceiling, as well as by reflections inside the system. At more favorable conditions, a maximum imaging range of up to 100 m could be achieved, which is determined by the receiver noise (a violet dashed line in Figure 5).

### 4.3. Angular Resolution at a Long Range

In Section 4.1 and Section 4.2, the angular resolution and the maximum imaging range were examined independently: the angular resolution was measured at a short range, and the maximum range was explored at a one-element receiving mode. In this section, the angular resolution and the maximum imaging range are examined simultaneously; an experiment on the distinction of two objects located at a long range was conducted.

**Figure 6:**
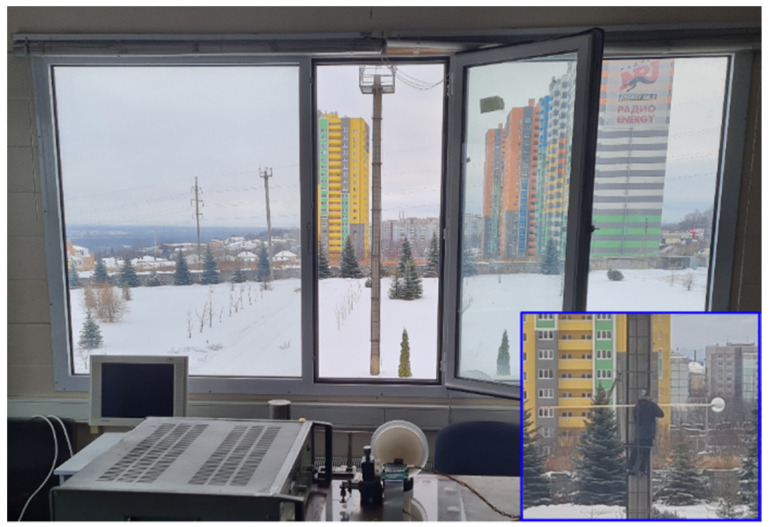
An overall photograph of the scene at an initial condition of the outdoor experiment: a pole without the spherical-cap reflector. Inset: a close-up photograph of reflecting objects; a spherical-cap reflector is being attached to the pole.

An overall photograph of an initial arrangement of the scene (without a spherical-cap reflector) is shown in Figure 6. The imaging system is located opposite to an open window on the second floor of the building. A single reflecting object that comes into the field of view of the imaging system is a pole with a diameter of 0.6 m. The distance from the imaging system to the pole is 44 m. The array receiver is positioned at the focal plane of the lens in the way that the maximum amplitude of the BF signal is achieved at the left (if we see from the scene, as in Figure 3) receiving element. No useful signals are at the other receiving elements at this position.

Then a spherical-cap reflector with the radius of the sphere of 60 cm and the diameter of the base of the cap of 30 cm was adjusted to the pole with a thin wooden beam (the inset of Figure 6). The reflector was moved from the pole with a step of 20 cm, and the amplitude spectrums of the BF signals of the receiving elements were recorded at each step. The initial spacing between the center of the pole and the center of the reflector was 50 cm, and the final spacing was 330 cm.

**Figure 7:**
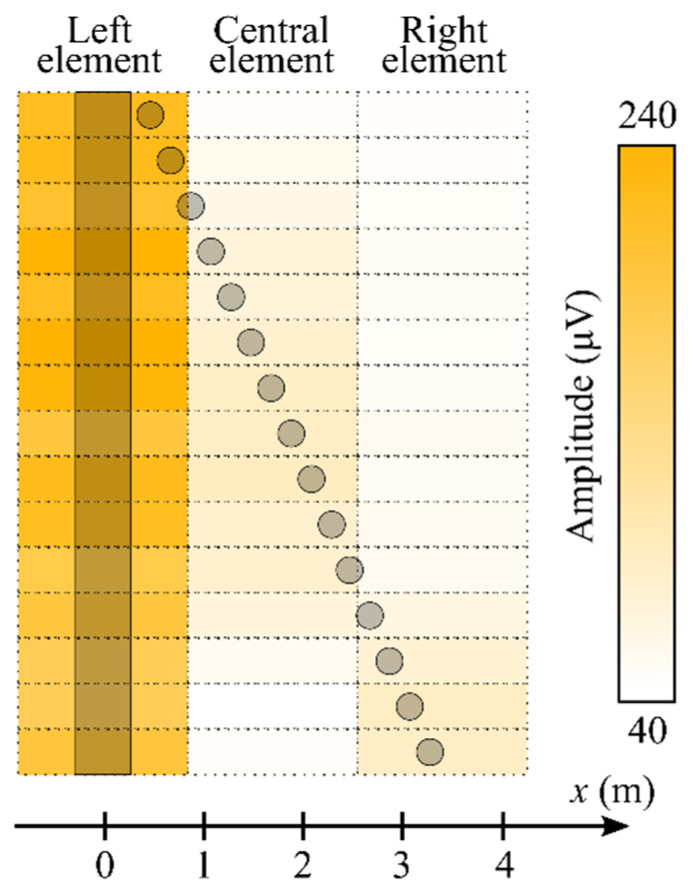
The amplitude of the BF signals of the left, the central, and the right receiving elements (the order is in correspondence to Figure 3) of the array receiver in dependence on different positions of the spherical-cap reflector (*x*). The dark bar is the pole, and the dark circles are the reflector at the different positions. The dotted cells mark space regions of the scene that correspond to fields of view of the receiving elements.

The amplitude of the BF signals of the receiving elements at each step of the reflector movement is shown in Figure 7 with a picture that clearly depicts a correspondence between a position of the reflector (*x*) and a response of the receiving elements. At an initial spacing, both the pole and the reflector are within a field of view of the left element. The signals at the central and right elements are determined by the clutter, which is caused by reflections inside the room, as well as reflections inside the system. The signal at the central receiving element emerges at the reflector position *x* = 110 cm, with the amplitude exceeding the clutter by about two times. The signal level from the reflector is two or three times lower than the signal from the pole, and this is due to the lower radar cross-section (RCS) of the spherical-cap reflector. At *x* = 270 cm, the amplitudes of the BF signal of the central and right elements become equal, and then the reflector comes completely to a field of view of the right element.

As seen in Figure 7, the pole and the spherical-cap reflector begin to become distinguishable at *x* > 270 cm. This value should be reduced by 30 cm for comparison with the result of the short-range experiment described in Section 4.1. The reducing is caused by the fact that the side areas of the pole also reflect the RF power, and the true spacing between two objects is not the spacing between their centers in this case. Thus, the minimum angle at which the pole and the spherical-cap reflector are distinguishable is *θ* = 3.2°, which coincides with the previously obtained short-range value.

## 5. 8 × 8 Array Imaging System

**Figure 8:**
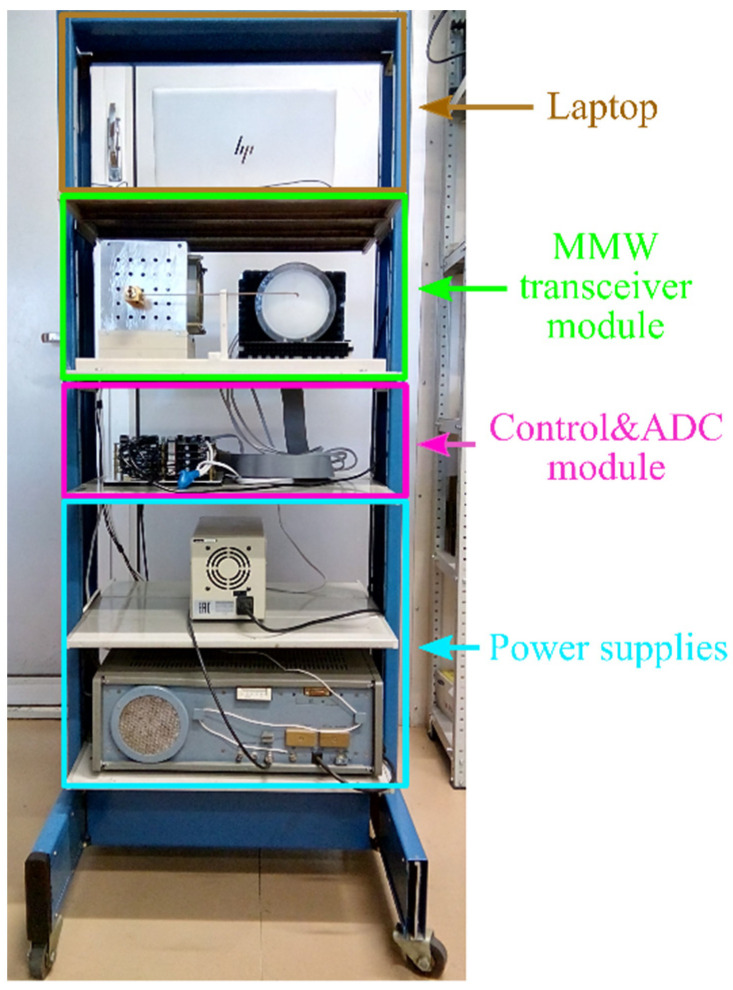
A photograph of the 8 × 8 array imaging system with designation of the modules, namely a MMW transceiver module, a control&ADC module, power supplies, and a laptop.

A photograph of the 8 × 8 array imaging system is presented in Figure 8. The system includes an MMW transceiver module, a control and analog-to-digital converter (ADC) module (control&ADC module), power supplies, and a laptop.

**Figure 9:**
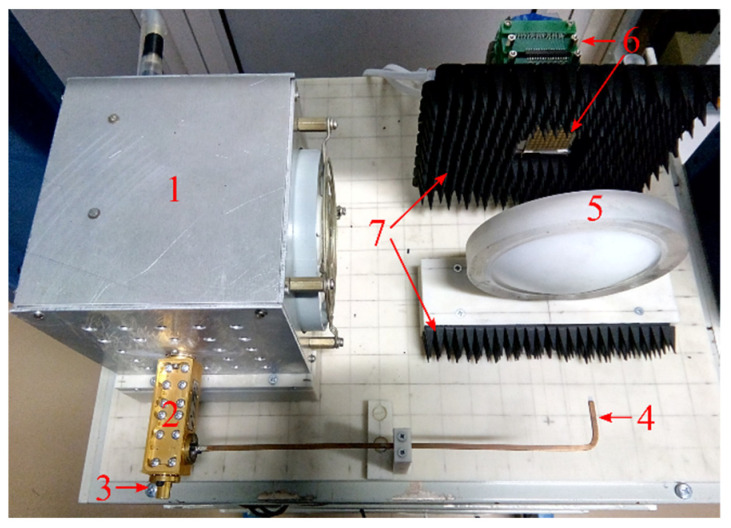
A photograph of the MMW transceiver module. (1) W-band BWO, (2) directional coupler, (3) horn antenna, (4) dielectric rod antenna, (5) dielectric lens, (6) array receiver, and (7) RF absorber.

The MMW transceiver module is shown in Figure 9. The OV-71 BWO with 10 mW output power is used for generating MMW signal, as in the 1 × 3 array imaging system. The triangular modulation waveform is used with a period of 10 ms and peak-to-peak amplitude of 14 V, which corresponds to a bandwidth of *B* = 672 MHz.

The output power of the BWO is divided into two equal parts with a Dok NO-10/3 directional coupler. The scene is illuminated with a Dok 92–96 GHz Prime Source conical horn antenna, which has a gain of 15 dB and a half-power beamwidth of 30° at the E-plane and 34° at the H-plane.

**Figure 10:**
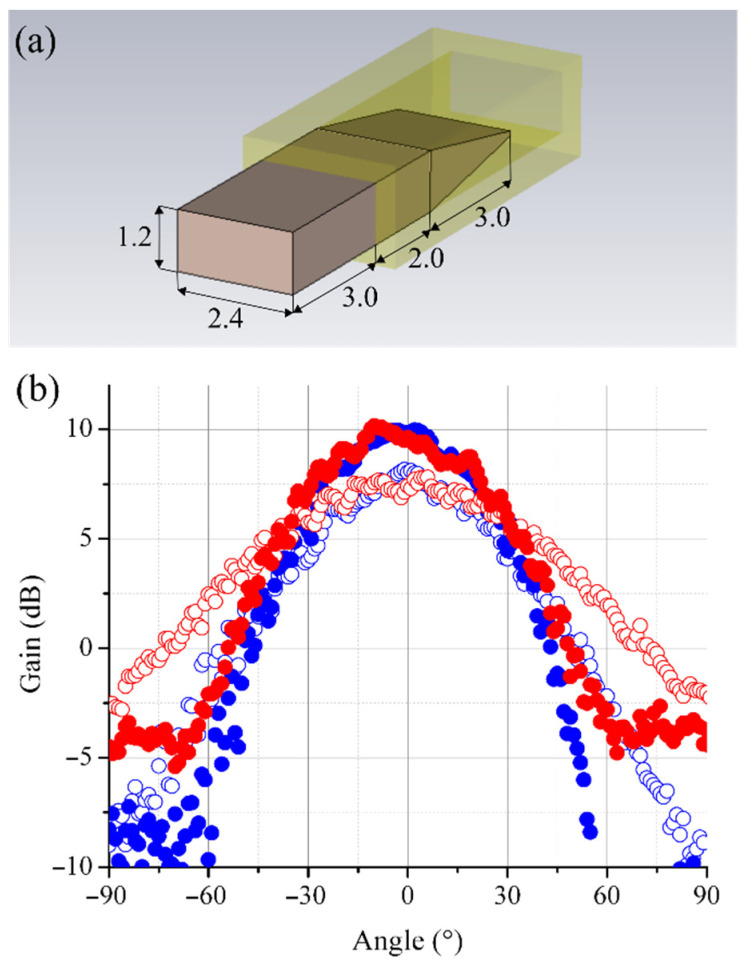
(**a**) A 3D model of the dielectric rod antenna, which is intended for providing the LO power to the array receiver. The dimensions are given in mm. (**b**) The gain of an open end of the waveguide (open circles) and the dielectric rod antenna (filled circles) at the E-plane (red circles) and at the H-plane (blue circles).

A dielectric rod antenna was developed for adapting a radiation pattern of the open end of the waveguide to a size of the objective aperture. The same 10 cm–diameter lens objective was used in the 8 × 8 array imaging system as in the 1 × 3 array one. A 3D model of the dielectric rod antenna with the main dimensions is presented in Figure 10a. The key element of the antenna is a polytetrafluoroethylene (PTFE) rod that is inserted into the 2.4 × 1.2 rectangular waveguide. The rod has small dimensions that ensure negligible perturbations of the received radiation falling onto the lens from the scene. The radiation pattern of the developed antenna is shown in Figure 10b (filled circles). A half-power beamwidth of 60° at the E-plane and 52° at the H-plane is obtained.

**Figure 11:**
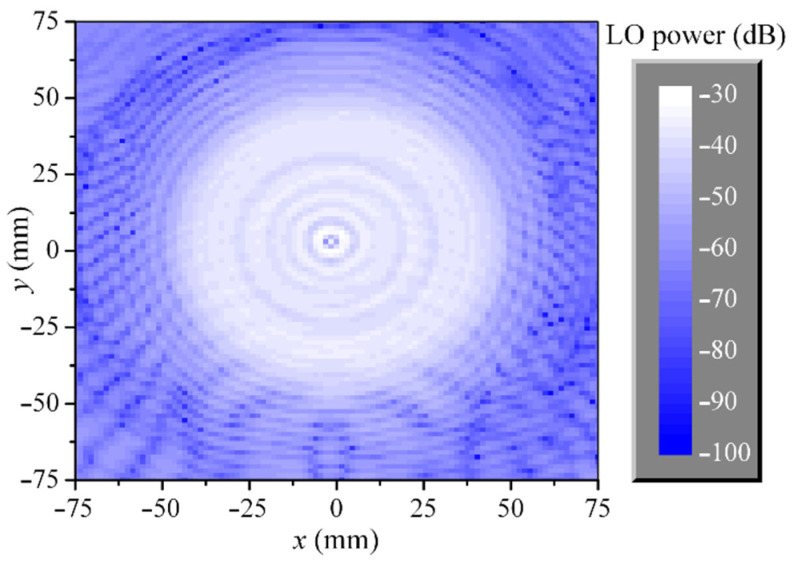
An LO power distribution at the focal plane (a view from the receiver side). In this experimental data, the LO power is the power measured with a probe antenna at the focal plane in relation to power fed to the dielectric rod antenna.

A smooth shape of the rod antenna radiation pattern does not ensure a smooth shape of the LO power distribution at the focal plane. For that reason, exploration of this distribution was conducted. A vector network analyzer (VNA) was used for measuring the S_21_ parameter, where the first port was the rod antenna, and the second port was a probe antenna moved at the focal plane. The probe antenna is an open end of the 2.4 × 1.2 rectangular waveguide with modified form of the outer walls for reducing scattering. The positioning accuracy of the probe antenna is 0.1 mm. The measured LO power distribution shown in Figure 11 indicates the presence of power inhomogeneity that achieves up to 27 dB in the region where the array is located. A possible reason for this inhomogeneity is related to cutting the rod antenna radiation pattern with the lens frame. The inhomogeneity could be reduced by narrowing the radiation pattern or by increasing the diameter of the lens.

A part of the LO power falls onto the area outside the array receiver. RF absorbers are incorporated into the design of the transceiver module to exclude unwanted reflections in the system. The RF absorbers are fabricated with graphite filled (25% by weight) acrylonitrile butadiene styrene (ABS) in fused filament fabrication (FFF) technology. The RF absorber consists of elements that are right square pyramids with a base length of 8 mm, a perpendicular height of 24–28 mm and a rotation angle around the height axis of −10° to 10°. The height and the rotation angle are set randomly for each pyramid. A reflection coefficient of −33 to −35 dB has been measured with a Michelson interferometer [35] in the 100–200 GHz range.

**Figure 12:**
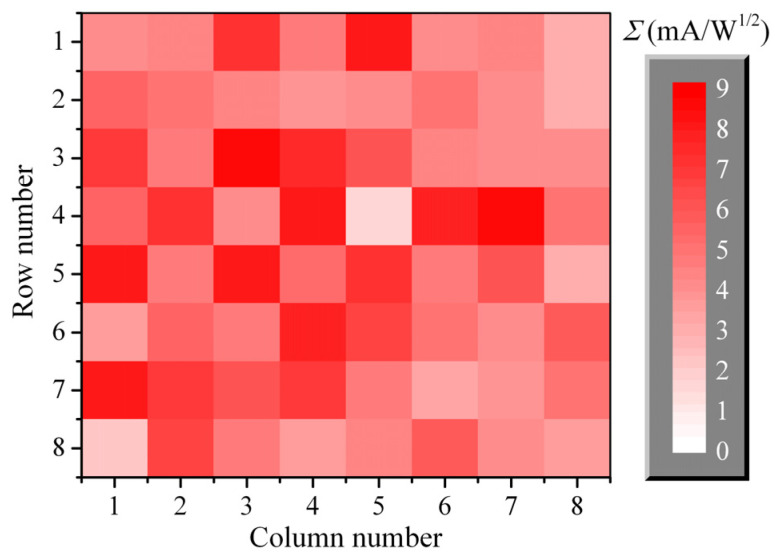
A map of the mixing elements sensitivity (*Σ*). The spatial arrangement of the map pixels corresponds to the spatial arrangement of the array pixels if we look at the receiver from the back side.

The low-barrier Mott diodes with a differential resistance of 3–7 kΩ and a curvature coefficient of 18–25 A/W were chosen for 8 × 8 array receiver fabrication. A stack of the four 16-channel BF amplifiers (see Section 3) was manufactured. The current sensitivity (*R_I_*) of the array elements, which can be used as detectors, was measured firstly; values of 0.8–3.0 A/W were obtained. The LO power incident on the mixing element (*P_LO_*) was measured by using the element itself as a detector; values of 0.6–4.5 μW were obtained. A dependence of *Σ* on *P_LO_* in the square-law region of the sensing element (*P_LO_* < ~10 μW) is given by the formula *Σ* = *Σ*_2_*P_LO_*^1/2^ [26]. The proportionality constant *Σ*_2_ is related to the current sensitivity (*R_I_*), as *Σ*_2_ = 2*R_I_* (see Appendix A). Therefore, the measured values of *R_I_* and *P_LO_* allow us to find the sensitivity of the mixing element, *Σ*. A map of *Σ* over the array elements is shown in Figure 12. The sensitivity of the elements varies from 2 to 9 mA/W^1/2^. Wide dispersion of the *Σ* values is caused by inhomogeneity both *R_I_* and *P_LO_*. The difference in sensitivity of the mixing elements is eliminated by introducing the proper correction coefficients in the signal-processing program.

The noise of the receiver includes the noise of the mixing element and the noise of the BF amplifier. The mixing-element noise is the thermal noise of the diode, which is defined by its differential resistance (*R_d_*). The BF amplifier noise has a more complex spectral density characteristic, which consists of a low-frequency flicker and higher-frequency broadband noise. Within the specified parameters of the imaging system, the BF is higher than the corner frequency (~1 kHz) of the amplifier, which allows us to consider only the frequency-independent broadband noise. The input-referred current and voltage noises of the BF amplifier are 3.1 pA/Hz^1/2^ and 5.7 nV/Hz^1/2^, respectively. The corresponding noise factor (*F_amp_*) at the *R_d_* = 3–7 kΩ input resistances is 3–5. The values of the total noise current (<*i_N_*^2^>^1/2^) of the receiving elements referring to the BF amplifier input lie within a range of 3.6–4.5 pA/Hz^1/2^. The noise spectral amplitude, *I_N_* = (2<*i_N_*^2^>)^1/2^, is a more convenient quantity when the amplitude spectrum of the BF signal is computed.

The NEP of the direct-conversion receiver based on a square-law mixing element is given by the formula *NEP* = (*I_N_*/*Σ*)^2^ [26]. The noise, *I_N_*, has low dispersion; thus, a map of *NEP* is similar to a map of *Σ* shown in Figure 12. The values of *NEP* are within a range of 3.9 × 10^−19^–9.2 × 10^−18^ W/Hz.

The smallest received power that can be detected by the developed imaging system prototype is increased due to a clutter BF signal that resulted in reflections of the LO power inside the system. The spectral amplitude of the clutter BF signal can exceed the noise spectral amplitude by one or two orders of magnitude, which can particularly be seen in Figure 5. The amplitude spectrum of the background signal *I_B_*(*f_b_*), which includes the receiver noise and the clutter signal, is well described by the function *I_B_* = *I_N_*(1 + (*f_b_*_0_/*f_b_*)^2^), where *f_b_*_0_ is a fitting parameter. Values of *f_b_*_0_ are different for different receiving elements and lie within a range from 60 kHz to 4 MHz. We suppose that the reflections that result in the clutter signal are from/inside the dielectric lens, which can lead to the law 1/*f_b_*^2^ of decreasing the clutter signal spectral amplitude at increasing *f_b_*. This issue, however, requires further research.

The control & ADC module consists of five stacked printed circuit boards (PCBs). The top PCB includes a function generator based on Texas Instruments TMS320F28377D micro controller unit (MCU) and a power supply for the BF amplifiers. An output waveform of the function generator has dc and ac components. The dc voltage sets the central frequency of the RF signal; the ac voltage serves as the modulation signal. The function generator signal passes to a high-voltage, high-power amplifier (a device at the lower shelf of the rack in Figure 8) that supplies power to the BWO. The function generator also provides a synchronization signal for the ADCs.

The bottom four PCBs are identical. Each of them contains four four-channel ADCs (16 ADCs in total) based on the TMS320F28377D MCUs. Thereby, there is an individual ADC for each receiving element, which provides digitizing, recording, and averaging of the BF signal. The linear increasing part of the triangular modulation signal is used to obtain the data.

The averaged digitized data are transmitted from the control & ADC module to a laptop, where the further signal processing is performed. The signal level is corrected in accordance with the sensitivity, *Σ*, of the corresponding receiving element. Then these signals undergo the FFT procedure, resulting in the amplitude spectrums of the BF signals for all receiving elements.

As is pointed out in Section 2, at a linear part of a *f*(*t*) dependence, the BF (*f_b_*) is proportional to the range to an object (*r*). Parameters *r_0_* and *k* of the relationship *r* = *r*_0_ + *kf_b_* should be determined in a calibration procedure. This procedure starts with measuring the experimental dependence *f_b_*(*r*), which is then approximated by the theoretical dependence *f_b_* = (*r* − *r*_0_)/*k*, with the fitting parameters *r_0_* and *k*. Measurements at distances from 1 to 16 m were carried out for calibrating the 8 × 8 imaging system. The values *r_0_* = 0.11 m and *k* = 0.998 m/kHz were obtained. The reference point of the distance is at the aperture of the horn antenna. It should be noted that, at the given system parameters, values of *f_b_* lie in the 100 kHz range up to a distance of about 100 m.

## 6. Demonstration Measurements

**Figure 13:**
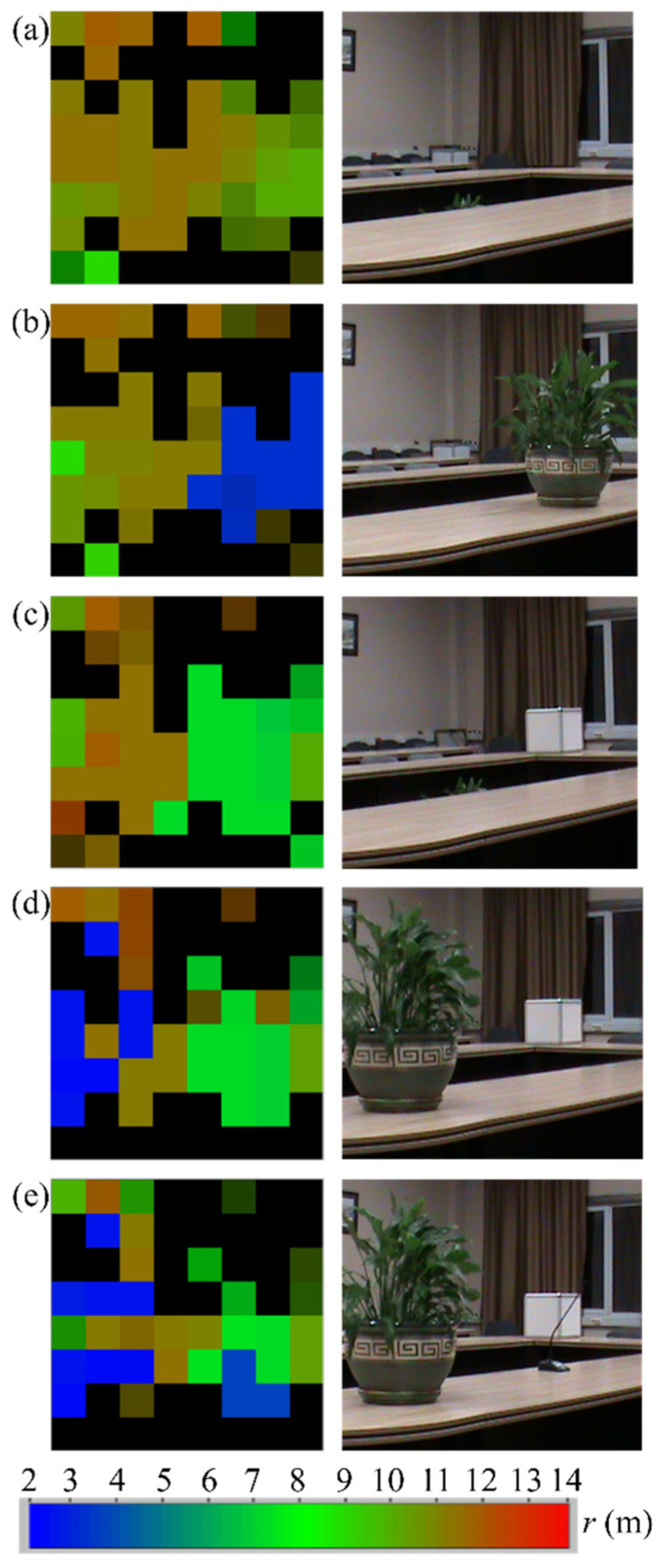
MMW images (**left**) and the related photographs (**right**) of a room region. The range to an object is indicated by color in accordance with a scale shown at the bottom of the figure. (**a**) An initial arrangement of the room. (**b**) A flowerpot is placed on the table. (**c**) The flowerpot is removed; a box is placed on the table. (**d**) The flowerpot is added toward the left. (**e**) A microphone is added.

The operation capability of the developed 8 × 8 array imaging system was demonstrated by imaging a room region with the presence and absence of different objects. The MMW images and the related photographs are shown in Figure 13. In order to obtain the MMW image, a spectral line with the maximum amplitude within a given BF range is determined for each pixel. A BF range that corresponds to a distance range from 2 to 14 m was chosen for obtaining images shown in Figure 13. The amplitude of the spectral line is coded by the color intensity of the image pixel. The distance is indicated by pixel color in accordance with a given color scale. If the amplitudes of the spectral lines are below the specified threshold, which is determined by the background signal, the image pixel is set to black.

An MMW image and the related photograph of the explored room region at an initial arrangement are shown in Figure 13a. A measured range to the wall is 10–12 m. It should be noted that we do not see a table at the bottom of the MMW image. This can be explained, firstly, by the mirror reflection of the transmitted RF power toward the wall, and, secondly, by the presence of a mirror image of the wall that is formed by the table surface. A flowerpot is placed on the table in Figure 13b. Then the flowerpot is removed, and a box is placed on the table (Figure 13c). The box has an image that is stretched out downward. This effect has the same source as the effect of table absence in Figure 13a: we see a mirror image of the box that is formed by the table surface. In Figure 13d, the flowerpot is placed on the left side of the frame. A microphone overlaps a part of the box in Figure 13e.

## 7. Conclusions

Millimeter waves are used in many applications, and their area of use constantly grows. One of the important applications is robotic systems, which are intended to replace humans in many difficult and dangerous scenarios. Optical cameras, which are actively used in robotic systems, have their limits, and other sensors are often necessary for image acquiring. The robotic systems that operate in bad conditions could be equipped with cameras of different frequency ranges, such as optical, infrared, terahertz, and MMW cameras. This would allow for us to obtain the most complete information about the environment. The optical cameras have high resolution but low penetration. The resolution of MMW cameras is not the highest, but millimeter waves can penetrate through fog, smoke, dust, etc. Cameras of various frequency ranges could give the robotic systems the ability that is not available to humans.

This work was devoted to creating an MMW imaging system with improved characteristics. A new method of acquiring an image in the MMW range was studied. This method is an evolution of the well-known FPA MMW imaging approach. The main difference is the use of a direct-conversion array receiver while employing an FMCW radar technique. This approach allows us to increase the maximum imaging range up to ~100 m, thus keeping the main characteristics of the FPA MMW imaging systems at the same level.

## Appendix A

The current–voltage characteristic *i*(*u*) of the receiving diode can be represented in the form of the Maclaurin series:

(A1) $$i\;=\;\sum_{n\;=\;1}^{\infty }a_{n}u^{n},$$

where *a_n_* (*n* = 1, 2, …) are the expansion coefficients. At a low level of the received power (in the square-law region of the diode),

(A2) $$\left|a_{n}u^{n}\right|>>\left|a_{n+1}u^{n+1}\right|,$$

and the first two terms of the series (A1) are responsible for detecting and mixing properties. Thus, we can write the following equation:

(A3) $$i\;=\;a_{1}u\;+\;a_{2}u^{2}.\;$$

Let us consider the direct detection mode of receiving the RF power. The RF voltage at the input of the diode (*u_RF_*) is given by the following expression:

(A4) $$u_{RF}\;=\;U_{RF}cos\left(\omega_{RF}t\right),$$

where *U_RF_* is the amplitude and *ω_RF_* is the angular frequency of the signal. After substituting (A4) to (A3) and keeping only a dc component of the output current, we find the following:

(A5) $$i_{dc}\;=\;\frac{a_{2}U_{RF}^{2}}{2}.$$

Now, let us consider the direct conversion mode of receiving the RF power. The LO voltage,

(A6) $$u_{LO}=U_{LO}cos\left(\omega_{LO}t\right),$$

is added to the RF voltage (A4), in this case, where *U_LO_* is the amplitude and *ω_LO_* is the angular frequency of the LO signal. By substituting a sum of *u_RF_* and *u_LO_* to (A3) and keeping only a BF component of the output current, we obtain the following:

(A7) $$i_{BF}\;=\;a_{2}U_{RF}U_{LO}cos\left[\left(\omega_{RF}\;-\;\omega_{LO}\right)t\right].$$

The input voltage amplitude is proportional to the square root of the input power:

(A8) $$U_{RF\left(LO\right)}\;=\;K\sqrt{P_{RF\left(LO\right)}},$$

where *K* is a constant coefficient that depends on the antenna parameters and efficiency of matching the antenna with the diode. By substituting (A8) into (A5) and (A7), we obtain the following:

(A9) $$i_{dc}\;=\;\frac{a_{2}K^{2}P_{RF}}{2}\;=\;R_{I}P_{RF},$$

(A10) $$\begin{array}{c} i_{BF}=a_{2}K^{2}\sqrt{P_{RF}P_{LO}}cos\left[\left(\omega_{RF}-\omega_{LO}\right)t\right]\\ =\Sigma_{2}\sqrt{P_{RF}P_{LO}}cos\left[\left(\omega_{RF}-\omega_{LO}\right)t\right], \end{array}$$

where we can define the following:

(A11) $$R_{I}\;=\;\frac{a_{2}K^{2}}{2},$$

(A12) $$\Sigma_{2}=a_{2}K^{2}\;$$

By comparing (A11) with (A12), we finally find the following:

(A13) $$\Sigma_{2}\;=\;2R_{I}.$$
